# Australian Lentil Breeding Between 1988 and 2019 Has Delivered Greater Yield Gain Under Stress Than Under High-Yield Conditions

**DOI:** 10.3389/fpls.2021.674327

**Published:** 2021-06-02

**Authors:** Victor O. Sadras, Garry M. Rosewarne, Lachlan Lake

**Affiliations:** ^1^South Australian Research and Development Institute, School of Agriculture, Food and Wine, The University of Adelaide, Adelaide, SA, Australia; ^2^Agriculture Victoria, Horsham, VIC, Australia

**Keywords:** crop growth rate, biomass, genetics, harvest index, phenology, phenotype

## Abstract

The contemporary lentil (*Lens culinaris* ssp. *culinaris*) industry in Australia started in the late 1980s. Yield in farmers’ fields averages 1.2 t ha^–1^ nationally and has not increased over three decades. Lack of yield progress can be related to a number of non-mutually exclusive reasons: expansion of lentil to low-yielding environments, lack of genetic gain in yield, lack of progress in agronomic practices, and lack of adoption of superior technologies. The aims of this study were to (i) quantify the genetic gain in lentil yield since 1988, (ii) explore the variation in the expression of genetic gain with the environment, and (iii) identify shifts in crop phenotype associated with selection for yield and agronomic adaptation. We grew a historic collection of 19 varieties released between 1988 and 2019 in eight environments resulting from the factorial combination of two sowing dates, two water regimes, and two seasons. Across environments, yield varied 11-fold from 0.2 to 2.2 t ha^–1^. The rate of genetic gain averaged 20 kg ha^–1^ year^–1^ or 1.23% year^–1^ across environments and was higher in low-yield environments. The yield increase was associated with substantial shifts in phenology. Newer varieties had a shorter time to flowering and pod emergence, and the rate of change in these traits was more pronounced in slow-developing environments (e.g., earlier sowing). Thermal time from sowing to end of flowering and maturity were shorter in newer varieties, and thermal time from pod emergence to maturity was longer in newer varieties; the rate of change in these traits was unrelated to developmental drivers and correlated with environmental mean yield. Genetic gain in yield was associated with increased grain number and increased harvest index. Despite their shorter time to maturity, newer varieties had similar or higher biomass than their older counterparts because crop growth rate during the critical period increased with the year of release. Genotype-dependent yield increased over three decades in low-yield environments, whereas actual farm yield has been stagnant; this suggests an increasing yield gap requiring agronomic solutions. Genetic improvement in high-yield environments requires improved coupling of growth and reproduction.

## Introduction

**FIGURE 1:**
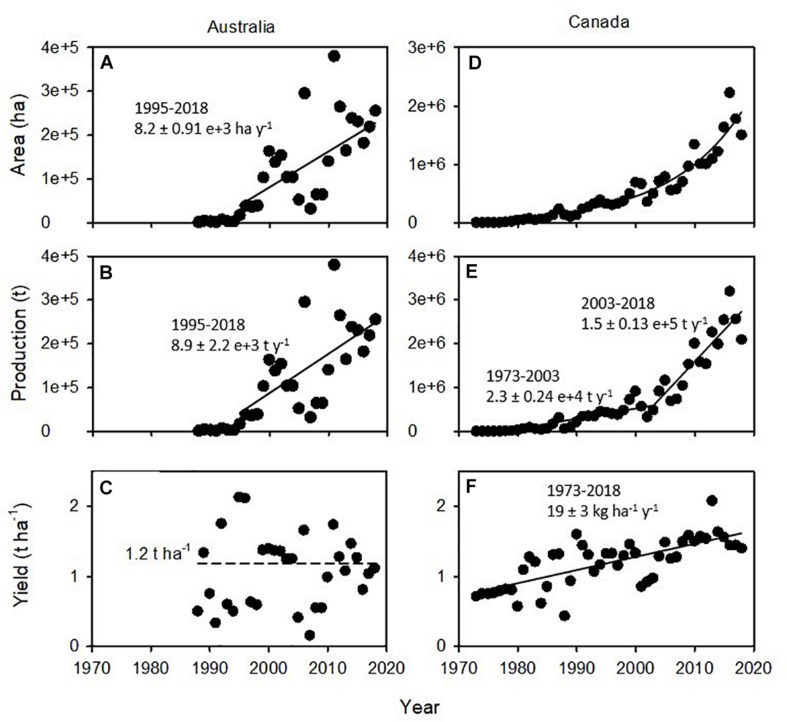
Area, production, and yield of lentils in **(A–C)** Australia and **(D–F)** Canada. In **(A,B,E,F)**, slopes and standard errors are shown for the fitted least-square regressions. In **(A,B,E)**, inflection points were identified fitting piece-wise models. Note difference in scales between Australia and Canada for area and production. Source: FAOSTAT, July 2020.

Australia currently produces over 300,000 t of lentils annually and contributes to approximately 10% of global trade, whereas Canada produces over 3 Mt and accounts for 50% of trade. The contemporary lentil industry in Australia started in the late 1980s with the introduction of late flowering, low-yielding forage types, and after a lag phase, acreage increased linearly since the mid-1990s (Figure 1A). Production increased in parallel to acreage (Figure 1B), whereas national average yield remained stagnant at 1.2 t ha^–1^, with large variation from failed crops to ∼2 t ha^–1^ (Figure 1C). In comparison, the acreage of the Canadian lentil industry grew exponentially since its inception, and increases in both acreage and yield contributed to an increase in production (Figures 1D–F).

Lack of progress in lentil average national yield in Australia can be related to several non-mutually exclusive reasons: expansion of the crop to drier, lower-yielding environments; lack of genetic improvement in yield; lack of progress in agronomic practices; and lack of adoption of superior technologies. Most of the Australian lentil is grown in the medium rainfall areas (350–450 mm year^–1^) of southern Australia, in particular, the sandy loam soils in South Australia and the alkaline gray cracking clays of Victoria. These regions feature winter-dominant rainfall, with a combination of drought, frost, and heat restricting the yield of pulses (Sadras et al., 2012; Lake et al., 2016, 2021). Supported by better agronomy (Llewellyn et al., 2012), pulses in the Mallee have increased from 7% in 2006 to 24% in 2017; this increase was at the expense of fallow, which declined from 18 to 2%, and pasture, which declined from 18 to 12% (Moodie and Brand, 2019). In comparison with the more productive Wimmera (440 mm year^–1^)^1^, where lentil yield can reach more than 4.5 t ha^–1^, yields in the Mallee (300 mm year^–1^)^2^ are up to ∼3.5 t ha^–1^. Hence, expansion of the crop into drier areas has likely contributed to stagnant national average yield. A strong focus on lentil herbicide tolerance to improve weed management may have also had indirect consequences for yield (Mao et al., 2015; McMurray et al., 2019).

Here, we focus on genetic improvement. Despite recognized limitations, retrospective studies comparing historic collections of varieties are routinely used with two objectives—to quantify the rate of genetic gain of a given breeding program and to uncover phenotypic changes associated with selection for yield (Austin et al., 1980; Slafer, 1994; Fischer et al., 2014; Tamagno et al., 2020). The assumption underlying the second objective is that making explicit the realized phenotypic change can guide further improvement. The absolute rate of genetic gain (kilograms per hectare per year) is often higher in environments with higher yield potential (Austin et al., 1980; Sadras et al., 2016), whereas the relative rate of genetic gain (percentage per year) is mostly independent of the environment (Fischer et al., 2014); quantifying the environmental influence on the expression of genetic gain in yield is thus important. The aims of this study were to (i) quantify the genetic gain in lentil yield since 1988, (ii) explore variation in the expression of genetic gain with the environment, and (iii) identify shifts in the crop phenotype associated with selection for yield and agronomic adaptation.

## Materials and Methods

### Experimental Design, Varieties, and Environments

We reanalyze the results of experiments reported by Lake and Sadras (2021), including 19 varieties released and used in the Australian lentil breeding program between 1988 and 2019 (Table 1). Crops were grown in eight environments with an 11-fold variation in yield from 0.2 to 2.2 t ha^–1^. Lake and Sadras (2021) emphasized yield components from a physiological perspective; here, we focus on yield and phenotypic shifts with the year of release.

**TABLE 1:** Seed type, phenology, and yield of 19 lentil varieties.

Variety^a^	Type	Year of release	Thermal time from sowing to (°Cd)				Yield (g m^–2^)
			Flowering	Pod emergence	End of flowering	Maturity	
Indianhead^b^	Red	1988	1546 ± 81.0	1679 ± 56.8	1940 ± 70.5	2193 ± 73.8	19 ± 6.9
Matilda	Green	1993	1273 ± 64.2	1374 ± 38.5	1706 ± 72.5	2034 ± 87.0	120 ± 17.1
Aldinga	Red	1995	1315 ± 68.7	1451 ± 42.0	1761 ± 68.0	2094 ± 76.4	129 ± 16.8
Northfield	Red	1995	1368 ± 80.9	1515 ± 51.9	1751 ± 64.9	2080 ± 78.6	129 ± 23.7
Nugget	Red	2000	1296 ± 70.0	1431 ± 45.3	1726 ± 67.0	2033 ± 84.4	99 ± 13.9
Boomer	Green	2008	1251 ± 53.3	1360 ± 34.8	1736 ± 68.3	2046 ± 78.9	101 ± 10.4
Nipper	Red	2008	1346 ± 78.9	1469 ± 46.6	1746 ± 68.7	2045 ± 82.0	128 ± 18.7
PBA Flash	Red	2009	1272 ± 58.3	1371 ± 36.9	1728 ± 66.2	2041 ± 76.3	140 ± 19.5
PBA Blitz	Red	2010	1096 ± 31.9	1236 ± 23.8	1602 ± 44.3	1969 ± 82.7	131 ± 14.5
PBA Jumbo	Red	2010	1275 ± 64.2	1396 ± 39.7	1722 ± 64.2	2022 ± 78.2	146 ± 22.7
PBA Ace	Red	2011	1208 ± 45.7	1321 ± 27.8	1717 ± 68.6	2008 ± 80.9	116 ± 14.2
PBA Bolt	Red	2011	1191 ± 44.3	1320 ± 27.6	1693 ± 61.3	2028 ± 80.3	141 ± 14.7
CIPAL0901^c^	Red	2013	1130 ± 38.5	1258 ± 26.7	1637 ± 55.1	1983 ± 85.6	153 ± 15.2
PBA Hurricane	Red	2013	1225 ± 45.6	1337 ± 32.8	1679 ± 59.7	2028 ± 77.0	124 ± 16.3
PBA Giant	Green	2014	1168 ± 42.5	1289 ± 28.2	1706 ± 66.7	2025 ± 77.7	97 ± 11.5
PBA Greenfield	Green	2014	1249 ± 49.9	1375 ± 33.0	1742 ± 64.7	2046 ± 76.1	110 ± 20.4
PBA Jumbo2	Red	2014	1216 ± 57.0	1344 ± 32.0	1734 ± 67.0	2013 ± 78.5	121 ± 13.6
CIPAL1504^c^	Red	2018	1239 ± 51.8	1369 ± 37.7	1753 ± 68.5	2056 ± 79.0	141 ± 25.8
CIPAL1701^c^	Red	2019	1106 ± 41.0	1238 ± 23.8	1676 ± 72.5	1963 ± 87.5	180 ± 22.5

*Values are BLUPs ± standard error across eight environments. ^*a*^Original study of Lake and Sadras (2021) comprised 20 varieties, including Commando. Here, we exclude Commando because it was not used in Australian breeding. ^*b*^Indianhead was an imported variety used extensively in the early stages of the breeding program (Inder et al., 2008). ^*c*^CIPAL lines have not been released as varieties but have been tested in National Variety Trials (NVT), the precursor stage to release. Year of release has been estimated for these lines based on the usual time spent in NVT. Idrissi et al. (2019) used a similar criterion to project the year of release of promising lentil lines in the Moroccan breeding program. BLUPs, best linear unbiased predictions.*

Trials were established on a calcic luvisol soil at Roseworthy (−34.5, 138.69). Briefly, environments resulted from the combination of two seasons (2018, 2019), two sowing dates, and two water regimes. Early sowings were on April 24, 2018, and April 29, 2019, and the late sowings on June 6, 2018, and June 24, 2019. Early-sown crops were irrigated or rainfed until June 26, 2018, and August 1, 2019, when rainout shelters were deployed to exclude rainfall until harvest, whereas late-sown crops were irrigated or rainfed. Hereafter, we refer to irrigated treatment as “wet” and rainfed and rainout shelter treatments as “dry.” Sowing date was assigned to the main plot, water regime to subplot, and varieties randomized within subplots with three replicates per treatment. Each experimental plot comprised six rows, 0.23 m apart, 5 m long, with a target plant density of 120 plants m^–2^.

### Phenology, Yield, Biomass, Crop Growth Rate, and Harvest Index

Crops were phenotyped for phenology, crop growth rate, yield, and its components: biomass, harvest index, grain number, and grain size.

We scored phenology twice weekly to determine the time from sowing (S) to 50% of the plants within the plot at flowering (F), pod emergence (PE), end of flowering (EoF), and maturity (M). Phenological stages are expressed on a thermal time scale with a base temperature of 0°C (Summerfield et al., 1985). The ratio PE-M:S-M was taken as a measure of the grain filling period in relation to the total cycle.

We measured biomass and crop growth rate non-destructively using the Canopeo app (Patrignani and Ochsner, 2015), which provides a two-dimensional measure of canopy coverage, combined with canopy height to return a three-dimensional trait. We used a calibration derived from a separate trial, in which we regressed actual biomass *vs*. Canopeo × height. Canopeo photographs were taken looking down from 140 cm every 7–10 days.

At maturity, we harvested shoots in 1-m^2^ sections from the four central rows of the plot to determine grain yield and its components. Harvest index was derived from shoot biomass and grain yield. Further details of methods are in Lake and Sadras (2021).

### Data Analysis

We tested trait response to variety, environment, and the interaction using analysis of variance with Genstat (20th edition). Best linear unbiased predictions were calculated with Multi Environment Trial Analysis with R for Windows version 6.0. We calculated the genetic rate of change as the slope of the least-square regression between trait and year of release. We calculated actual rates, e.g., kilograms per hectare per year for yield, and rates relative to the newest variety (Fischer et al., 2014). Rates were calculated for data pooled across all environments and for each environment separately. Environmental dependence in the expression of genetic shifts in yield and other traits was explored by plotting the rate of genetic change against the environmental mean of yield and the environmental mean of the trait. We report *p*-value as a continuous quantity and Shannon information transform [*s* = -log_2_(*p*)] as a measure of the information against the tested hypothesis (Greenland, 2019).

## Results

### Growing Conditions

Table 2 summarizes growing conditions and yield in the eight environments. Growing-season rainfall + irrigation ranged from 117 mm for the early-sown, dry crop in 2018, to 332 mm for the early-sown, wet crop in 2019. Across varieties, yield ranged from 21 g m^–2^ for early-sown, dry treatment in 2018, to 221 g m^–2^ for early-sown, wet treatment in 2018. Across varieties, average yield was positively associated with growing season rainfall (*y* = −18.1 + 0.59 *x*, *R*^2^ = 0.50; *p* = 0.052, *s* = 4.3) and with minimum temperature (*y* = −90.8 + 38.2 *x*, *R*^2^ = 0.69; *p* = 0.010, *s* = 6.6).

**TABLE 2:** Mean yield across varieties and growing conditions in eight environments resulting from combinations of season, sowing date, and water regime.

Season	Sowing date	Water regime	Yield (g m^–2^)	Seasonal rainfall + irrigation (mm)	Daily average for the critical period					Thermal time from sowing to (°Cd)			
						
					Tmax (°C)	Tmin (°C)	Radiation (MJ m^–2^)	VPD (kPa)	PTQ (MJ m^–2^°C^–1^)	*F*	PE	EoF	*M*
2018	Early	Wet	221	289	16.9	7.1	9.9	0.7	0.8	1411	1575	2318	2835
	Early	Dry	21	117	16.9	4.9	12.7	0.8	1.2	1337	1467	1670	2008
	Late	Wet	110	240	21.6	4.5	18.2	1.3	1.5	1167	1280	1570	1827
	Late	Dry	77	203	21.7	4.1	18.8	1.4	1.6	1160	1282	1537	1769
2019	Early	Wet	145	332	20.8	5.9	14.8	1.2	1.2	1451	1585	2069	2362
	Early	Dry	71	156	18.4	3.8	14.5	1.0	1.4	1408	1519	1740	2057
	Late	Wet	131	324	23.5	6.2	19.7	1.6	1.5	1039	1131	1350	1582
	Late	Dry	183	218	25.6	7.6	19.4	1.8	1.3	1127	1223	1533	1840

*Tmax is maximum temperature, Tmin is minimum temperature, VPD is vapor pressure deficit, and PTQ is photothermal quotient. Critical period was between flowering and 200°Cd after flowering, averaged across varieties.*

### Phenology

All phenostages varied with variety, environment, and their interaction (Supplementary Table 1). Table 3 shows absolute and relative rates of change of phenological traits for the pooled data. Across environments, thermal time from sowing to flowering, pod emergence, end of flowering, and maturity were all shortened with the year of release. In contrast, the thermal time between pod emergence and maturity and the proportion of the season between pod emergence and maturity both increased with the year of release.

**TABLE 3:** Absolute and relative rate of genetic change (±SE) for lentil traits in varieties released between 1988 and 2019.

Trait	Absolute	Relative (% year^–1^)
Yield	20 ± 6.9 kg ha^–1^ year^–1^	1.23 ± 0.28
Thermal time sowing to flowering	−9 ± 1.6°Cd year^–1^	−0.78 ± 0.08
Thermal time sowing to pod emergence	−4.9 ± 1.7°Cd year^–1^	−0.72 ± 0.08
Thermal time sowing to end of flowering	−4.9 ± 2.9°Cd year^–1^	−0.27 ± 0.05
Thermal time sowing to maturity	−4.5 ± 3.6°Cd year^–1^	−0.22 ± 0.04
Thermal time pod emergence to maturity	4.9 ± 2.6°Cd year^–1^	0.56 ± 0.13
Ratio thermal time pod emergence-maturity/sowing-maturity	0.003 ± 0.0007 year^–1^	0.73 ± 0.11
Crop growth rate	0.07 ± 0.02 kg ha^–1^°Cd^–1^ year^–1^	1.46 ± 0.35
Biomass	16 ± 21 kg ha^–1^ year^–1^	0.38 ± 0.15
Harvest index	0.004 ± 0.001 year^–1^	1.25 ± 0.25
Grain number	34 ± 18 seeds m^–2^ year^–1^	0.92 ± 0.31
Grain size	0.40 ± 0.08 mg seed^–1^ year^–1^	0.96 ± 0.20

*Rates are the slope of least-square regressions between trait and year of release for data pooled across eight environments. Relative rate is percentage of the latest variety.*

**FIGURE 2:**
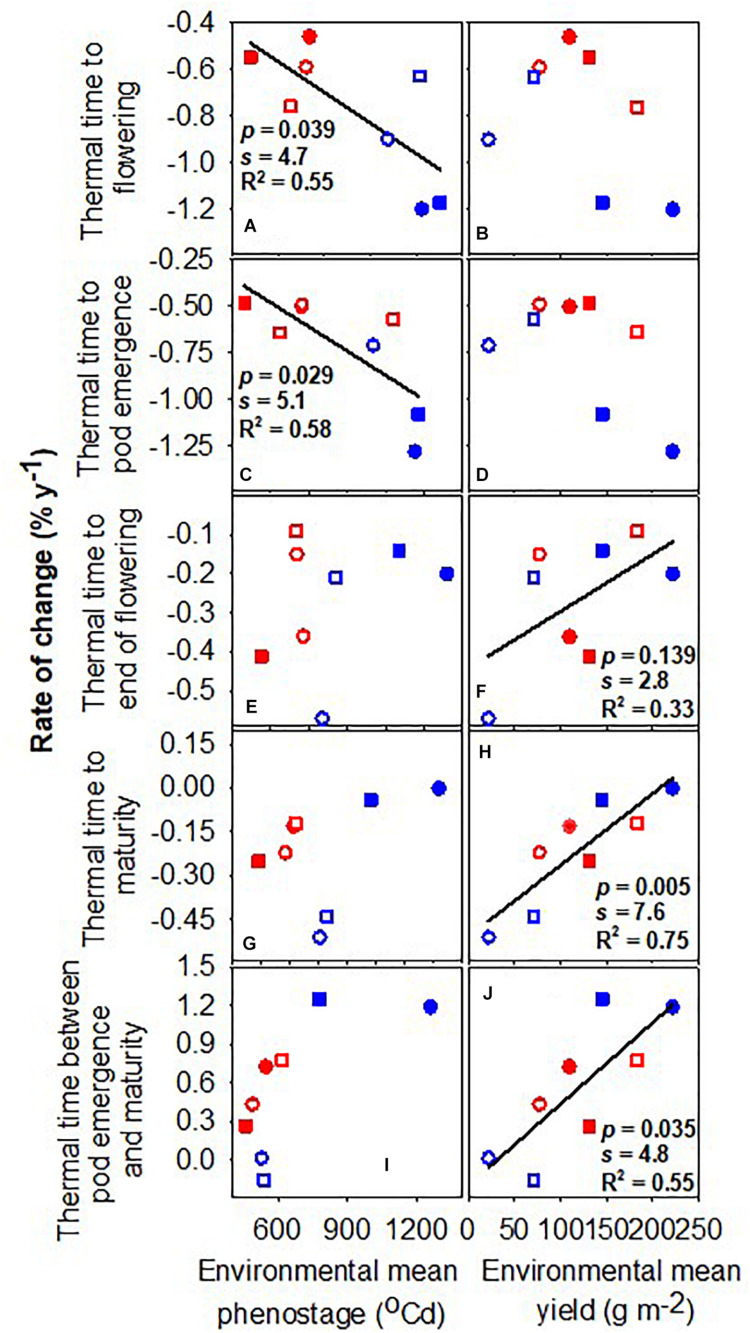
Rate of change of thermal time from sowing to flowering, pod emergence, end of flowering and maturity, and the duration between pod emergence and maturity against the environmental mean phenostage **(A,C,E,G,I)** and the environmental mean yield **(B,D,F,H,J)**. Lines are least-square regressions and are only presented where *p* < 0.05, *s* > 4.3. Rates are relative to the newest variety. Symbols are: blue (2018), red (2019), circles (early sowing), square (late sowing), open (rainfed), closed (irrigated).

**FIGURE 3:**
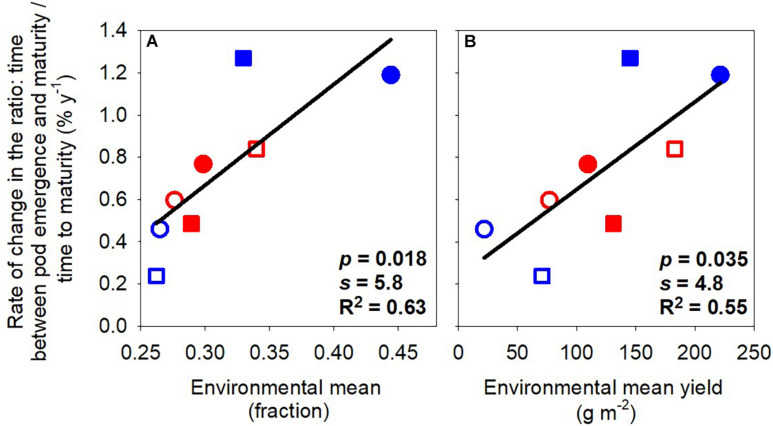
Rate of change of the ratio: time between pod emergence and maturity/time to maturity against environmental mean of the ratio **(A)** and environmental mean yield **(B)**. Lines are least-square regressions. Rates are relative to the newest variety. Symbols are: blue (2018), red (2019), circles (early sowing), square (late sowing), open (rainfed), closed (irrigated).

Figure 2 shows the rate of change of phenological traits with the year of release as a function of (a) the environmental mean for the trait and (b) the environmental mean for yield. The environmental mean of the trait captures temperature, photoperiod, and water influences on development, empirically defining slow- and fast-developing environments. For example, the environmental mean thermal time to flowering ranged from 1039°Cd in the late-sown wet treatment 2019 to 1451°Cd in the early-sown wet treatment in 2019 (Table 2). The rates of change in thermal time to flowering and to pod emergence were stronger, i.e., more negative, in environments favoring slower development (Figures 2A,C). For example, the rate of change in flowering changed from −1.20 to −0.46% year^–1^ with environmental means from 1411 to 1167°Cd. The rates of change in thermal time to flowering and maturity were proportional to environmental mean yield (Figures 2F,H,J) and unrelated to the environmental mean of the phenostage (Figures 2E,G,I). Thermal time from pod emergence to maturity relative to thermal time from sowing to maturity was related to the environmental mean for both duration of phenostage and yield (Figure 3).

### Yield and Its Components

**FIGURE 4:**
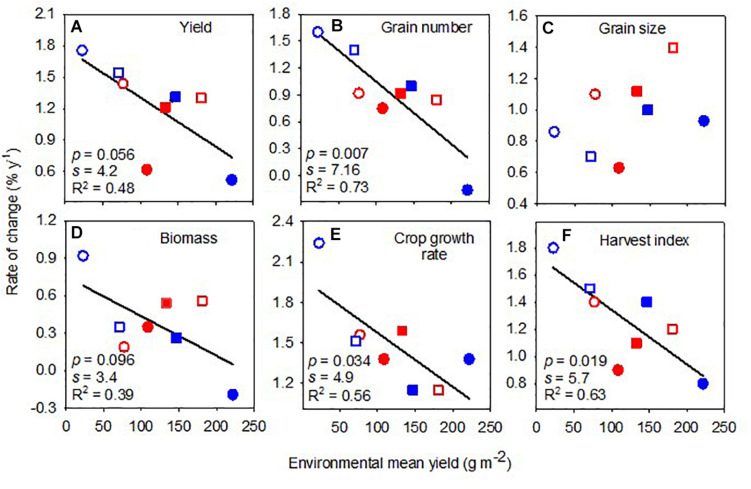
Rate of change of yield **(A)**, grain number **(B)**, grain size **(C)**, biomass **(D)**, crop growth rate **(E)**, and harvest index **(F)** against environmental mean yield. Lines are least-square regressions. Rates are relative to the newest variety. Symbols are: blue (2018), red (2019), circles (early sowing), square (late sowing), open (rainfed), closed (irrigated).

Yield varied ninefold with variety (Table 1) and 10-fold with environment (Table 2), with no interaction between environment and variety (Supplementary Table 1). Across environments, yield increased with the year of release at 20 kg ha^–1^ year^–1^ or 1.23% year^–1^ (Table 3). The rate of genetic gain in yield declined linearly with increasing environmental mean yield (Figure 4A).

Grain number varied fourfold with variety and 10-fold with the environment, with a significant interaction between environment and variety (Supplementary Table 1). Across environments, grain number increased with the year of release at 34 seeds m^–2^ year^–1^ or 0.92% year^–1^ (Table 3). The rate of change in grain number with the year of release was higher in low-yielding environments (Figure 4B). Grain size varied with variety (twofold) and with the interaction between environment and variety (Supplementary Table 1). Across environments, grain size increased by 0.40 mg seed year^–1^ or 0.96% year^–1^ (Table 3). The rate of genetic change in grain size was unrelated to environmental mean yield (Figure 4C).

Shoot biomass at maturity varied little between varieties (<1.5-fold) and varied ∼5-fold with environment, with no interaction between environment and variety (Supplementary Table 1). Across environments, the absolute rate of change in biomass with the year of release was close to zero, and the relative rate was 0.38% year^–1^ (Table 3). The association between the relative rate of change in biomass and environmental mean yield was weak and negative (Figure 4D).

The crop growth rate in the critical period varied 2.5-fold with variety and fourfold with the environment, with no interaction between environment and variety (Supplementary Table 1). Across environments, the crop growth rate increased with the year of release at 0.07 kg ha^–1^°Cd^–1^ year^–1^ or 1.46% year^–1^. The rate of change in crop growth rate with the year of release was higher in more stressful environments (Figure 4E).

Harvest index varied sixfold with variety and 3.5-fold with the environment and also varied with the interaction between environment and variety (Supplementary Table 1). Across environments, the harvest index increased 0.0042 year^–1^ or 1.25% year^–1^ (Table 3). The rate of increase in harvest index with the year of release almost halved between the lowest and highest yielding environments (Figure 4F).

## Discussion

### Genetic Gain in Yield Was Stronger in Stressful Environments

Our measured genetic gain for Australian lentils between 1988 and 2019 averaged 20 kg ha^–1^ year^–1^ or 1.23% year^–1^ across eight environments. It compares with the rate of 18–27 kg ha^–1^ year^–1^ for Ethiopian lentil in two environments (Bogale et al., 2015); 31–35 kg ha^–1^ year^–1^ for Moroccan lentil (Idrissi et al., 2019); 11–17 kg ha^–1^ year^–1^ for kabuli (Tadesse et al., 2018), and 32 kg ha^–1^ year^–1^ for desi chickpea in Ethiopia (Bekele et al., 2016).

Contrary to the observation that relative rates of genetic gain are independent of the environment in cereals (Fischer et al., 2014), here, we found that the expression of genetic gain in lentil yield was stronger under stress and often close to zero in high-yielding environments (Figure 4A). The rates of genetic change in the main drivers of yield, including grain number, crop growth rate, and harvest index, were also larger in low-yielding environments (Figure 4). Consistent with our finding, well-managed National Variety Trials in southern Australia, which benchmark current and new germplasm, show no improvement in either maximum or environmental mean yield between 2009 and 2018 (Supplementary Table 1). For lentils in Ethiopia, the rate of genetic gain in yield relative to the newest variety was 0.80% year^–1^ in an environment of 1.3 t ha^–1^ average yield and 0.92% year^–1^ in an environment of 4.8 t ha^–1^ (Bogale et al., 2015). For lentils in Morocco, the rate of genetic gain relative to the local check was 0.68% year^–1^ in a dry environment (200–350 mm year^–1^) compared with 1.0% year^–1^ in a wetter environment (300–500 mm year^–1^). We conclude that the proposition of environment-independent relative rates of genetic gain cannot be generalized.

The higher rate of genetic gain in low-yielding environments partially associates with the late phenology of early introductions. The breeding program has continually decreased time to flowering, podding, and maturity (Figure 2 and Table 3) as earliness is critical for yield in short, dry seasons (Silim et al., 1993; Kumar et al., 2012). Similarly, breeding has focused on taller and more upright crops to facilitate improved machine harvest in drier environments with actual gains of 0.12 cm year^–1^ (data not shown); lower crop growth rate and shorter plants of earlier varieties would impact yield under dry or short-season conditions (Erskine, 2009; Muehlbauer et al., 2009).

### Higher Proportion of Time From Pod Emergence to Maturity, Higher Harvest Index, and Higher Crop Growth Rate in the Critical Period Offset Earlier Flowering and Maturity

Genetic gain in yield in Mediterranean, East Asian, and Sub-Saharan African environments has been associated with earlier flowering in lentils, chickpea, and wheat (Siddique et al., 1989; Erskine et al., 1994; Berger et al., 2004, 2006; Sadras and Lawson, 2011; Bogale et al., 2015). This is an important adaptation, achieving yield before the concurrent water and thermal stress later in the season (Thomson et al., 1997; Erskine et al., 2011).

We found three traits that offset the reduction in yield associated with shorter time to flowering and maturity: a longer period from pod emergence to maturity relative to crop duration (sowing to maturity), an increased harvest index, and an increased growth rate during the critical period. Harvest index was partially related to the extended period from pod emergence to maturity.

In indeterminate lentil, early flowering, combined with a lengthening of the reproductive period, increases the probability of grain set and filling to occur in favorable conditions while maintaining vegetative growth. However, a lengthening of the reproductive period may have negative effects under extreme stress, with Syrian research showing reproductive duration was negatively associated with lentil yield (Silim et al., 1993). For our set of varieties and environments, there was a negative association between time to flowering and time between pod emergence and maturity in the longer duration environments, with no relationship in the stress environments (Supplementary Table 2). This is a reflection of the later flowering, earlier Australian releases being adapted from material originating in longer season environments where they can flower later and extend reproduction.

### Genetic Gain in Yield Primarily Associated With Growth Rate, Grain Number, and Harvest Index

The average rate of genetic gain in yield, 1.23% year^–1^, compares with the rate of change of 1.46% year^–1^ for growth rate, 0.92% year^–1^ for grain number, and 1.25% year^–1^ for harvest index. In soybean, early gains in yield were driven by increased biomass and harvest index (Koester et al., 2014; Suhre et al., 2014), and allometric analysis further highlights the improvement in reproductive allocation (Tamagno et al., 2020). Lentil can grow large dense canopies and tend to suffer from a low harvest index, particularly in higher-yielding conditions (Kusmenoglu and Muehlbauer, 1998; Hanlan et al., 2006; Lake and Sadras, 2021). Phenotypes adapted to the main producing regions of Canada are assumed to combine moderate biomass and high harvest index (Hanlan et al., 2006). Averaged across environments, CIPAL 1701 had the highest harvest index at 0.33, and the average across varieties was 0.23 compared with reported maxima 0.44–0.59 (Whitehead et al., 2000; Malhi et al., 2007; Unkovich et al., 2010); the maximum for our dataset (0.54) indicates an opportunity for improvement.

Grain size in Canadian lentil (Muehlbauer, 1974) and kabuli chickpea in India (Gowda et al., 2011) was negatively correlated with yield. Australian breeding between 1988 and 2019 has achieved both increased grain size and yield (Table 3). In United States soybean improvement, grain size increased initially (Specht and Williams, 1984), but more recent work shows grain number has driven yield gain (Tamagno et al., 2020); this is also the case for Canadian soybean (Voldeng et al., 1997); and Ethiopian common bean (Bezaweletaw et al., 2006).

### Trait Combinations Are Feasible

The indeterminate nature of lentils provides opportunities and challenges with large environmental variation in biomass. As biomass has low heritability, selection for crop growth rate in physiologically meaningful windows and harvest index are likely to be effective in increasing yield (Lake and Sadras, 2021). In short-season Mediterranean environments, combining early flowering and longer reproductive duration may improve harvest index and reduce problems associated with excessive vegetative growth. Successfully combining these traits may provide genetic gains in yield with less risk of a trade-off between yield in high- and low-yielding environments. Selection for early flowering is desirable in shorter Mediterranean environments, but there is a limit to how far flowering can be advanced against frost risk in the target population of environments (Lake et al., 2021). A longer flowering window can offset yield losses from limited frosts, but regular frosts may be more problematic, particularly in shorter seasons.

## Conclusion

Over the three decades of Australian lentil breeding and for our sample of varieties and environments, genetic gain in yield was 20 kg ha^–1^ year^–1^ or 1.23% year^–1^. The estimated genetic gain in yield was larger in lower-yielding environments. This genetic gain combined with improved agronomy has allowed the spread of lentils into lower rainfall regions of Australia, increasing rotational options and allowing more diverse cropping systems (Llewellyn et al., 2012; Moodie and Brand, 2019). The lack of improvement in the national average yield over this period is partially related to the expansion of the crop to intrinsically lower-yielding environments. Further improvements in lentil production require the adoption of improved practices to close the gap between water-limited and actual yield and a stronger focus in breeding for superior combinations of crop growth rate, biomass, and harvest index for higher yield potential.

## Data Availability Statement

The raw data supporting the conclusions of this article will be made available by the authors, without undue reservation.

## Author Contributions

VS contributed to the planning and analysis of research and the writing of the manuscript. GR contributed to the writing of the manuscript. LL contributed to the planning, performing, analysis of research, and the writing of the manuscript. All authors contributed to the article and approved the submitted version.

## Conflict of Interest

The authors declare that the research was conducted in the absence of any commercial or financial relationships that could be construed as a potential conflict of interest.

## Footnotes

1 http://www.climatekelpie.com.au/wp-content/uploads/2019/10/040-Wimmera-VIC-Climate-Guide.pdf

2 http://vro.agriculture.vic.gov.au/dpi/vro/vrosite.nsf/pages/rainfall#:∼:text=Median%20annual%20rainfall%20ranges%20from,parts%20of%20the%20mountainous%20regions
