# Supplementary figures and images for: Australian Lentil Breeding Between 1988 and 2019 Has Delivered Greater Yield Gain Under Stress Than Under High-Yield Conditions
Source: Front Plant Sci. 2021 Jun 2;12:674327. doi: 10.3389/fpls.2021.674327 (PMC8207196; doi:10.3389/fpls.2021.674327)

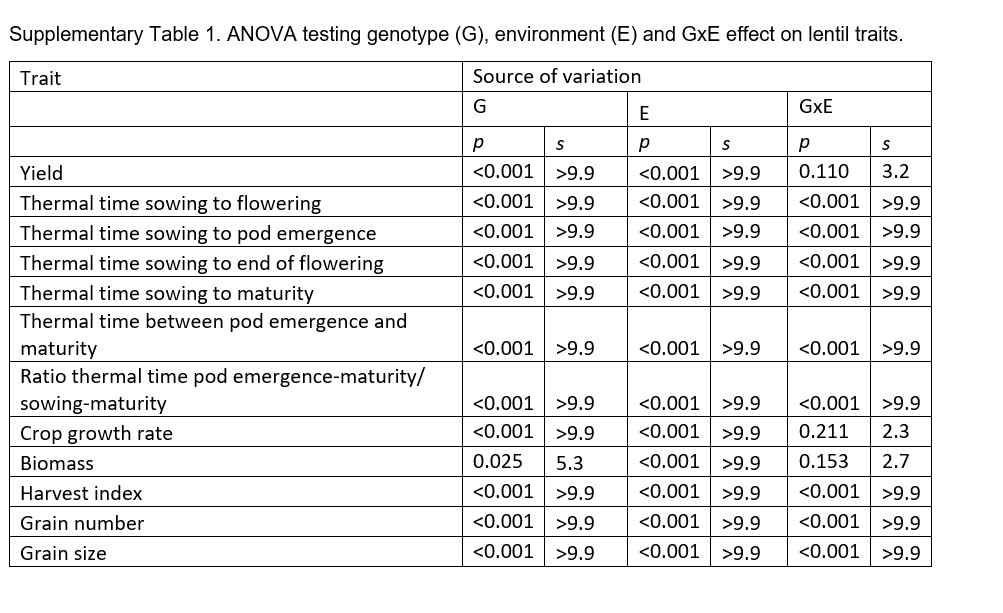

Supplement: Supplementary file 1 [file Data_Sheet_1.zip › Sup Table 1.jpg]

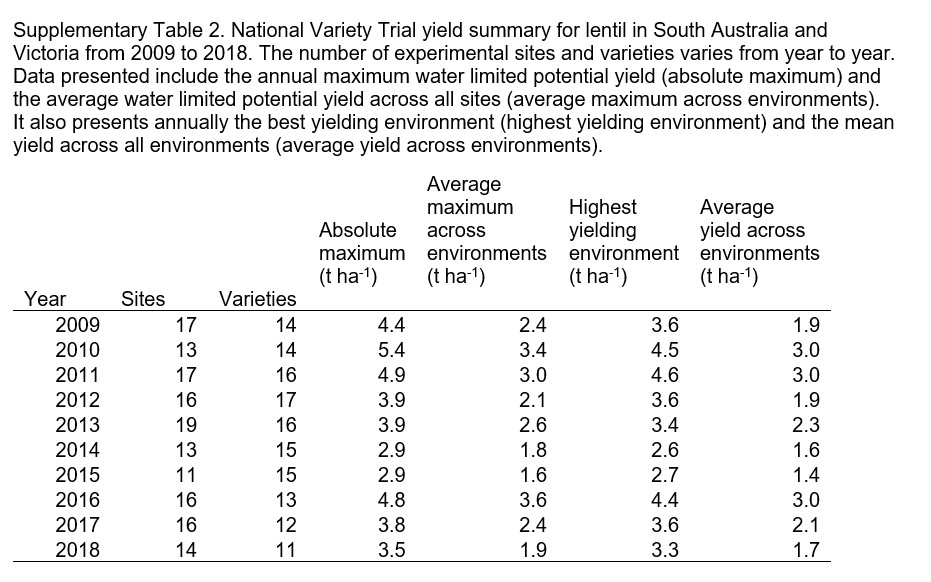

Supplement: Supplementary file 1 [file Data_Sheet_1.zip › Sup Table 2.jpg]
